# Treatment of intermittent hypoxia increases phosphorylated tau in the hippocampus via biological processes common to aging

**DOI:** 10.1186/s13041-016-0282-7

**Published:** 2017-01-05

**Authors:** Sosuke Yagishita, Seiya Suzuki, Keisuke Yoshikawa, Keiko Iida, Ayako Hirata, Masahiko Suzuki, Akihiko Takashima, Kei Maruyama, Akira Hirasawa, Takeo Awaji

**Affiliations:** 1 Department of Pharmacology, Faculty of Medicine, Saitama Medical University, 38 Moro-hongo, Moroyama-machi, Iruma-gun, Saitama 350-0495 Japan; 2 Faculty of Health and Medical Care, Saitama Medical University, 1397-1 Yamane, Hidaka-shi, Saitama 350-1241 Japan; 3 Department of Genomic Drug Discovery Science, Graduate School of Pharmaceutical Sciences, Kyoto University, 46-29 Yoshida Shimoadachi-cho, Sakyo-ku, Kyoto 606-8501 Japan; 4 Department of Life Science, Faculty of Science, Gakushuin University, 1-5-1 Mejiro, Toshima-ku, Tokyo 171-8588 Japan; 5 Present address: Department of Peripheral Nervous System Research, National Institute of Neuroscience, National Center of Neurology and Psychiatry, 4-1-1 Ogawa-higashi, Kodaira, Tokyo 187-8502 Japan

**Keywords:** Sleep-disordered breathing, Intermittent hypoxia, Gene ontology-based microarray analysis, Alzheimer disease, Tau

## Abstract

Sleep-disordered breathing produces cognitive impairments, and is possibly associated with Alzheimer disease (AD). Intermittent hypoxia treatment (IHT), an experimental model for sleep-disordered breathing, results in cognitive impairments in animals via unknown mechanisms. Here, we exposed mice to IHT protocols, and performed biochemical analyses and microarray analyses regarding their hippocampal samples. In particular, we performed gene ontology (GO)-based microarray analysis to elucidate effects of IHT on hippocampal functioning, which were compared with the effects of various previously-reported experimental conditions on that (ref. Gene Expression Omnibus, The National Center for Biotechnology Information). Our microarray analyses revealed that IHT and aging shared alterations in some common GO, which were also observed with kainic acid treatment, Dicer ablation, or moderate glutamate excess. Mapping the altered genes using the Kyoto Encyclopedia of Genes and Genomes PATHWAY database indicated that IHT and aging affected several pathways including “MAPK signaling pathway”, “PI3K-Akt signaling pathway”, and “glutamatergic synapse”. Consistent with the gene analyses, in vivo analyses revealed that IHT increased phosphorylated tau, reflecting an imbalance of kinases and/or phosphatases, and reduced proteins relevant to glutamatergic synapses. In addition, IHT increased phosphorylated p70 S6 kinase, indicating involvement of the mammalian target of rapamycin signaling pathway. Furthermore, IHT mice demonstrated hyperactivity in Y-maze tests, which was also observed in AD models. We obtained important data or something from the massive amount of microarray data, and confirmed the validity by in vivo analyses: the IHT-induced cognitive impairment may be partially explained by the fact that IHT increases phosphorylated tau via biological processes common to aging. Moreover, as aging is a major risk factor for AD, IHT is a novel model for investigating the pathological processes contributing to AD onset.

**Electronic supplementary material:**

The online version of this article (doi:10.1186/s13041-016-0282-7) contains supplementary material, which is available to authorized users.

## Introduction

Sleep-disordered breathing (SDB) is characterized by recurrent arousals from sleep and intermittent hypoxemia. Associations have been suggested between SDB and various disorders, including hypertension [1, 2], cardiovascular disease [3], diabetes [4], and dementia such as Alzheimer disease (AD) [5–7]. Patients with SDB often have cognitive impairments, which are associated with intermittent hypoxia, but not with sleep fragmentation or duration of sleep [8–10]. Thus, intermittent hypoxia treatment (IHT) has been used as an experimental animal model for SDB [11–23]. Consistent with previous clinical studies, rats or mice exposed to IHT, but not to sustained hypoxia [23], demonstrated memory impairment [16, 21–23], which was attenuated by exogenous administration of growth hormone or erythropoietin [12, 23]. However, the mechanisms of IHT-induced cognitive impairments have not been elucidated. In the present study, in order to find a clue to the mechanisms, we aimed to understand the effects of IHT in the hippocampus, which has crucial roles in learning and memory.

Various complex pathways are involved in cognitive impairments, which are often associated with aging and dementia such as AD [24], therefore, we first performed a microarray analysis. The resulting data were subjected to a principal component analysis (PCA), and gene ontology (GO)-based microarray analysis according to a previous report [25]. We analyzed not only our experimental data, but also various other data published in Gene Expression Omnibus (GEO: The National Center for Biotechnology Information), in relation to measure of hippocampal functioning. Comparing the massive amount of microarray data led to successful identification of several experimental models that shared gene alterations common with our IHT model. Next, we mapped the altered genes using the Kyoto Encyclopedia of Genes and Genomes (KEGG) PATHWAY database, and investigated several pathways that may be affected by IHT. Finally, we confirmed the insights obtained from in silico analyses by performing in vivo analyses, with findings indicating that IHT increases level of phosphorylated tau (P-tau).

## Methods

### Animals

C57BL/6J male mice were purchased from Tokyo Laboratory Animals Science Co., Ltd. (Tokyo, Japan), and began experiments when they were at 10-weeks-old. We also prepared mice reared for 12 months, in order to perform the microarray analysis. Animals were handled according to procedures approved by the Animal Care and Use Committee of Saitama Medical University.

### Antibodies

For immunoblots of total tau, we used rabbit polyclonal anti-tau, JM [26]. For immunoblots of phosphorylated tau at each epitope, we used the following antibodies: mouse monoclonal AT270 (Innogenetics, Gent, Belgium), which recognizes pThr181; rabbit polyclonal anti-pSer199 (Biosource, Camarillo, CA); mouse monoclonal AT8 (pSer202, pThr205) (Innogenetics); rabbit polyclonal anti-pThr212 (Biosource); rabbit polyclonal anti-pSer214 (Invitrogen, Carlsbad, CA); mouse monoclonal AT180 (Innogenetics), which recognizes pThr231; rabbit polyclonal anti-pSer396 (Biosource); rabbit polyclonal anti-pSer400 (Sigma-Aldrich, St. Louis, MO); rabbit polyclonal anti-pSer404 (Millipore, Billerica, MA). For immunoblots for other proteins, we used the following antibodies: mouse monoclonal anti-VGluT1 (Millipore); mouse monoclonal anti-synaptophysin (Sigma-Aldrich); rabbit polyclonal anti-GluA1 (Synaptic Systems, Goettingen, Germany); mouse monoclonal anti-GluA2 (Millipore); mouse monoclonal anti-PSD-95 (BD Transduction Laboratories, San Jose, CA); rabbit polyclonal anti-PICK1 (Thermo Fishier Scientific, Waltham, MA); mouse monoclonal anti-α-Tubulin (Sigma-Aldrich); mouse monoclonal anti-GSK-3β (BD Transduction Laboratories); rabbit polyclonal anti-phospho-GSK-3β (Ser9) (Cell Signaling Technology, Danvers, MA); mouse monoclonal anti- phospho-GSK-3β (Tyr216) (BD Transduction Laboratories); rabbit polyclonal anti-PP2A A subunit, B subunit, and C subunit (Cell Signaling Technology); rabbit polyclonal anti-p70 S6 kinase (Cell Signaling Technology); rabbit polyclonal anti-phospho-p70 S6 kinase (Thr389) (Cell Signaling Technology).

### Intermittent hypoxia treatment (IHT)

**Fig. 1:**
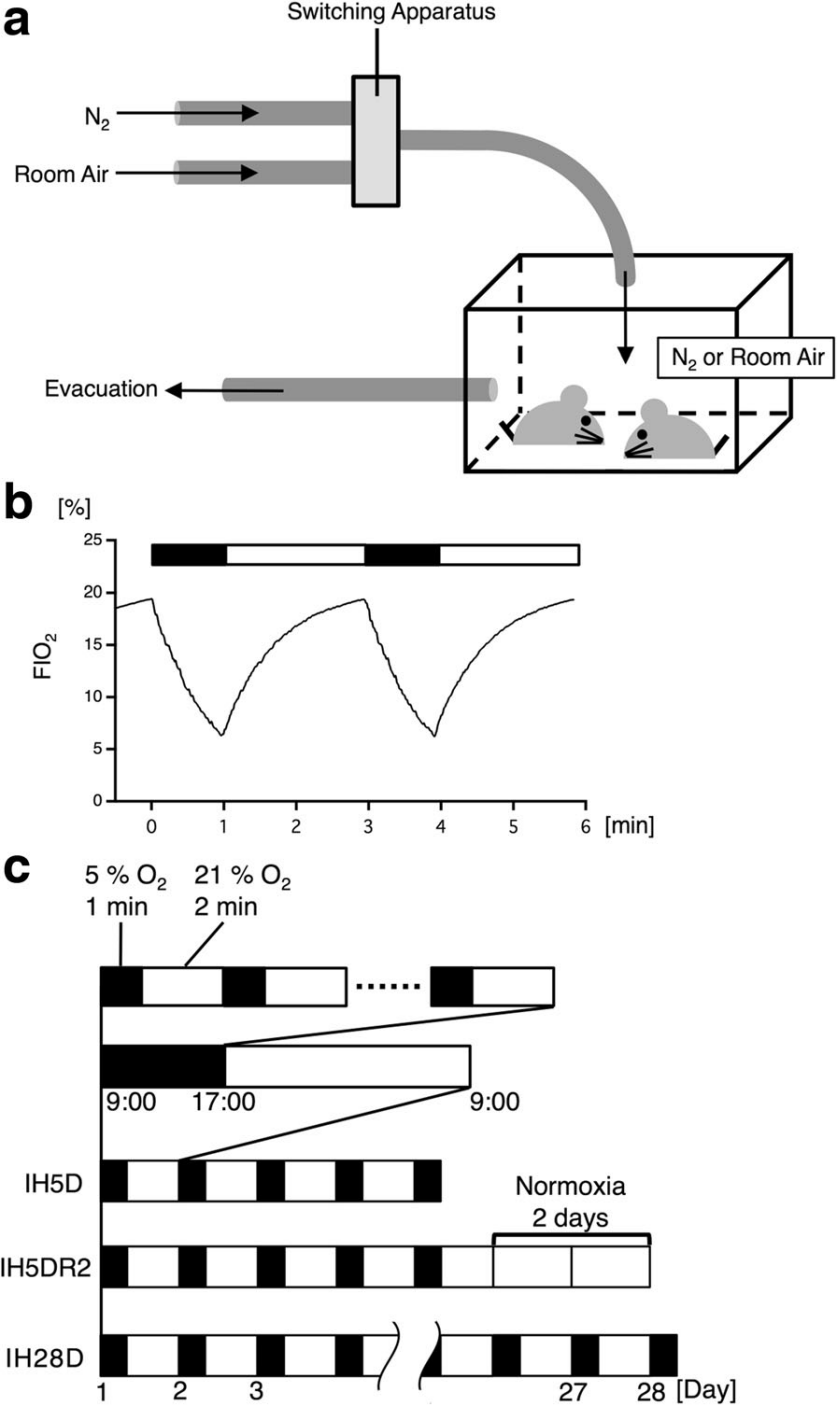
Intermittent hypoxia treatment (IHT) protocols. **a** The apparatus for IHT was composed of a chamber, in which mice were habituated, connected with an injection tube and an evacuation tube. The injection tube was connected to bottled N_2_ gas and an air pump through a switching apparatus with a solenoid valve and timers, by which gas was controlled for injection into the chamber. **b** Time course of fraction of inspired O_2_ (FIO_2_) during IHT. FIO_2_ was reduced to 5% by 1 min of pure N_2_injection, and FIO_2_ was increased to 21% by 2 min of room air injection. The black box indicates the duration of N_2_ injection, and the white box indicates the duration of room air injection. **c** The IHT protocol was composed of 3 min cycles, which included of 1 min of pure N_2_ injection followed by 2 min of room air injection. Cycles were repeated from 9:00 to 17:00. The remainder of the day (16 h) was composed of normoxia treatment. The IH5D group was exposed to IHT for 5 days, and the IH5DR2 group was exposed to IHT for 5 days, followed by normoxia treatment for 2 days. The IH28D group was exposed to IHT for 28 days. The black box indicates repetition of the 3 min cycles for 8 h. The white box indicates normoxia treatment

Mice were placed in a breeding cage connected to an apparatus that controlled gas injection (N_2_ or room air) and an evacuation tube (Fig. 1a). The IHT group was exposed to a protocol of 1 min of pure N_2_ injection, in order to reduce the fraction of inspired O_2_ (FIO_2_) from 21 to 5%, followed by 2 min of room air injection to increase FIO_2_ from 5 to 21% (Fig. 1b). This 3 min cycle was repeated for 8 h each day (from 9:00 to 17:00): we refer the duration as “IHT”. During the remaining 16 h, the animals were maintained in normoxia (from 17:00 to 9:00 in the next morning).

Mice underwent various protocols of IHT: IH5D, IH5DR2, and IH28D (Fig. 1c). Mice in the IH5D group were exposed to the IHT for 5 times, and then sacrificed immediately after the end of the final IHT. Mice in the IH5DR2 group were exposed to IHT for 5 times and followed by normoxia treatment for 64 h (16 h + 24 h × 2), and were then sacrificed. Mice in the IH28D group were exposed to the IHT for 28 times, and then sacrificed immediately after the end of the final IHT.

We applied the IH5D protocol as a standard to investigate the effect of IHT. The effects of rescue from IHT were investigated by exposing mice to the IH5DR2 protocol. The effects of elongating the IHT period were studied by exposing mice to the IH28D protocol. As the experimental control for the IH28D group, we reared mice for 28 days under normoxia condition (Ctrl28).

### Brain isolation

Immediately following the indicated protocol, mice were sacrificed by cervical dislocation according to procedures approved by the Animal Care and Use Committee of Saitama Medical University. We performed sacrifices and dissection in separate rooms. Isolated brains were chilled with phosphate-buffered saline, and stored at −80 °C until use.

### RNA extraction

Total RNA was extracted from hippocampus using ISOGEN (Nippon Gene, Tokyo, Japan), and was used for microarray analyses.

### Microarray analysis

Microarray analysis was performed as described previously [27]. Briefly, genome-wide mRNA expression profiles were obtained by microarray analysis with the Affymetrix GeneChip Mouse (430 2.0) Array, according to the manufacturer’s instructions. Normalized data are available at the GEO database (http://www.ncbi.nlm.nih.gov/geo/) under accession number GSE62385.

The microarray data obtained from the in vivo model and GEO (GSE32583 and GSE32591… etc.) were examined and visualized using R/Bioconductor [28], as described previously [25, 28]. The expression levels of our microarray data were calculated from probe intensities and arrays normalized using the mas5 method [29]. Only probe sets with normalized signals >20 were defined as expressed, and were selected for analyses. The expression of individual genes was defined as altered when comparison of the average normalized signal intensities using the Bioconductor package Genefilter gave a value with *P* <0.05 using Welch’s ANOVA. We performed principle component analysis on the results of standardized microarray data using the statistical package R. In order to investigate the ontology of differentially expressed genes, enriched expression of GO terms were assessed and confirmed with the hypergeometric test [30], using the Bioconductor package GOstats/GSEABase. These programs determine which GO terms identified from lists of affected genes are statistically over— or under-represented, compared to the GO terms represented in the microarray as a whole. Hierarchical clustering was performed using Ward’s method to calculate the linkage distances based on the correlation coefficient between samples or GO terms.

### Biochemical analysis

Hippocampal samples were homogenized with Tris-buffered saline (TBS) containing protease inhibitor cocktail (Roche Diagnostics Ltd, Mannheim, Germany) and phosphatase inhibitor cocktail (Nacalai Tesque, Kyoto, Japan). The homogenates were ultracentrifuged at 32,000 x *g*, for 20 min, at 4 °C (Rotor: TLA45, Beckman Coulter). Protein concentrations of the supernatants and the sonicated pellets were determined using a BCA protein assay kit (Nacalai Tesque), and were adjusted to 1 ~ 2 μg/μL with sample buffer containing SDS. Samples were boiled at 96 °C for 10 min before being used for conventional SDS-PAGE, followed by transfer to nitrocellulose membranes. After blocking with skim milk (MEGMILK SNOW BRAND Co., Ltd., Tokyo, Japan), the membranes were incubated with the indicated primary antibody. After treatment with horseradish peroxidase-conjugated secondary antibody, the membranes were treated with reagent for exposure (Chemi-Lumi One Super, Nacalai Tesque; ImmunoStar LD, Wako, Osaka, Japan). Images of the membranes were captured with the ChemiDoc^TM^ XRS+ system (Bio-Rad, Hercules, CA) or a C-Digit Blot Scanner (LI-COR, Lincoln, NE), and analyzed using Image J software.

### Y-maze test

The Y maze apparatus (Hazai-ya, Tokyo, Japan) was a 3-arm maze with equal angles between all arms (8 cm width) and a bottom with 40 cm (length) and 15 cm height. Mice were tested individually by placing them in an arm of the maze and allowing them to move freely throughout the 3 different arms for 10 min. The sequence and entries into each arm were recorded. An alternation was determined from successive consecutive entries into the 3 different arms on overlapping triads in which all arms were represented. For example, ACBABCABAB, a sequence of entries to the 3 arms A, B, or C, would generate 5 ‘successful’ alternations, ACB, CBA, ABC, BCA, and CAB; the total number of possible alternations corresponded to the number of the total arm entries minus 2 (in this example, the total number would equal 8). The percentage alternation was calculated as (the number of ‘successful’ alternations divided by the number of the total arm entries minus 2) × 100. We analyzed the percentage alternation and the total number of arm entries. The schedule of behavioral tests is shown in Fig. 5a or d. Mice completed the Y-maze test (Pre) and were then divided into control (Ctrl) and IHT groups. Significant differences between the 2 groups did not occur for the percentage alternation or the total number of arm entries (Figs. 5b and c, Pre; 5e-f, Pre). For the IH5D protocol (Fig. 5a), the IHT group was exposed to IHT for 5 days. For the remaining 2 days, all mice were treated with normoxia. At day 5 and 7, all mice completed the Y-maze test. For the IH28D protocol (Fig. 5d), the IHT group was exposed to IHT for 28 days. At day 28, all mice completed the Y-maze test.

### Statistical analysis

Statistical analyses were performed using Prism 6 (GraphPad Software Inc., La Jolla CA). Quantification of Western blotting data were analyzed by one-way ANOVA followed by Dunnett’s tests. We used Student’s t-tests to compare 2 groups. Analyses of behavioral data were performed by two-way ANOVA followed by Tukey’s multiple comparison tests. Statistical significance was indicated by *p* <0.05.

## Results

### GO-based microarray analysis reveals common biological processes between IHT and aging

In order to understand the effects of IHT on hippocampal functioning, mice underwent various IHT protocols: IH5D, IH5DR2, and IH28D (Fig. 1; also see the Methods section). As the experimental control for IH28D, we used mice reared for 28 days under normoxia condition (Ctrl28). Since a previous review indicated that IHT caused aging processes [31], we also studied mice reared for 12 months (12 m) at our institution. Total hippocampal RNA was extracted, and used for the microarray analysis.

**Fig. 2:**
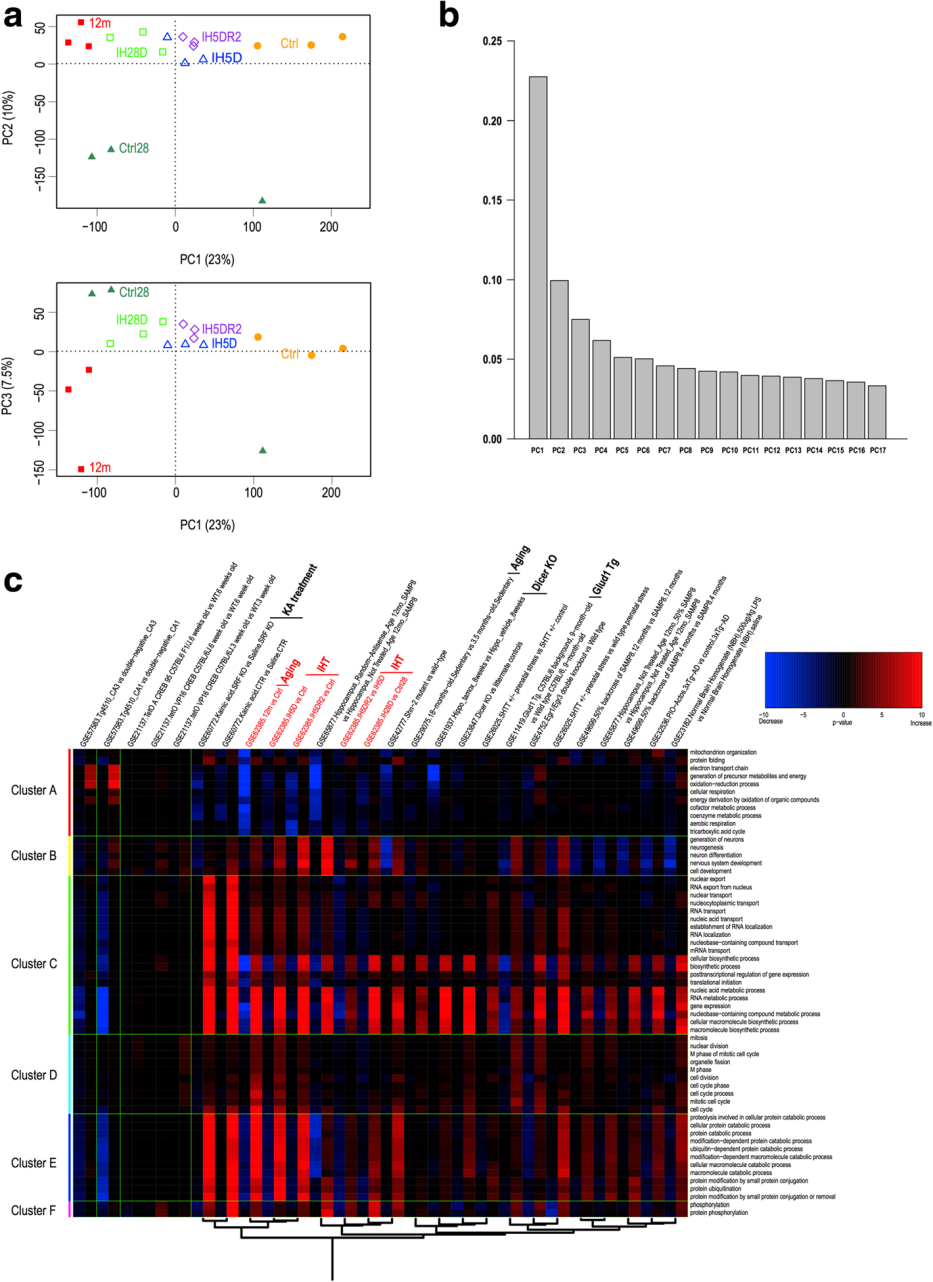
Principal component analysis (PCA) and partial results of the gene ontology (GO)-based microarray analysis. **a** PCA of hippocampal gene expression profiles. PCA plots show the 2 principle components with the greatest variation, which cover 23% (PC1) and 10% (PC2) (upper panel), or 23% (PC1) and 7.5% (PC3) (bottom panel) of the total variance, respectively. Symbols indicate the PCA representing profiles of control and experimental animals, consisting of 3 independent GeneChip experiments. **b** Screeplots of PCA results. **c** Portions of the complete heatmap (Additional file 1: Figure S1) generated by GO-based microarray analysis. The parts with green rectangles in Additional file 1: Figure S1 are extracted and displayed. GO terms are given on the vertical axis, and samples (comparisons of experimental conditions with their controls) are given on the horizontal axis. A dendrogram regarding the samples is shown at the bottom. In particular, our experimental data (GSE62385) are indicated with red-colored letters. *P* values were calculated by the hypergeometric test and denoted by -log_10_ *P*; overrepresented GO terms are shown in red and blue (increased (+) and decreased (−), respectively). Abbreviations: Ctrl = control group, IH5D = exposed to IHT for 5 days, IH5DR2 = exposed to IHT for 5 days, followed by normoxia treatment for 2 days, Ctrl28 = reared at our institution for 28 days, IH28D = exposed to IHT for 28 days, 12 m = reared at our institution for 12 months

We performed PCA, in order to assess global variance in gene expression profiles among control (Ctrl), IH5D, IH5DR2, Ctrl28, IH28D, and 12 m groups (Fig. 2a and b). The Ctrl and 12 m groups were located furthest along the PC1 axis, and the IH5D, IH5D2R, and IH28D groups were appeared in order at the middle of the axis (Fig. 2a). In addition, the Ctrl28 group was also appeared between Ctrl and 12 m along the PC1 axis.

Simultaneously, we performed a GO-based microarray analysis to compare our experimental data (GSE62385) with the various data obtained from GEO: hippocampal microarray data that were relevant to memory formation, cognitive impairment, neurodegeneration, and development. Based on the GO-based microarray analysis, we generated a GO heatmap with a gene clustering dendrogram on the left, indicating linkage in alteration profile. We also produced a sample correlation dendrogram on the bottom of the heatmap, indicating similarity in comprehensive gene expression among data from various experimental models (Additional file 1: Figure S1, the complete heatmap). Fig. 2c shows portions of the complete heatmap, which are marked with green rectangles in Figure S1. The dendrogram (Fig. 2c, bottom) revealed that similar alterations in gene expression patterns occurred with IHT (IH5D vs. Ctrl, IH5DR2 vs. Ctrl, IH28D vs. Ctrl28; GSE62385) and aging (12 m vs. Ctrl; GSE62385). Therefore, our PCA and GO-based microarray analyses indicate that IHT and aging shared common biological processes.

By using the heatmap, we focused on GO term clusters A-F that were altered by IHT or aging (Fig. 2c; Additional file 1: Figure S1): cluster A that was related to “energy production”, clusters B that was related to “neurogenesis”, clusters C that was related to “RNA metabolism”, clusters D that was related to “cell cycle”, clusters E that was related to “ubiquitination”, and clusters F that was related to “phosphorylation”. In particular, we summarized the alteration patterns of GO term clusters A, B, C and E (Table 1).

**Table 1:** Summary of GO-based microarray analysis

Experimental models		GO term clusters				GSE #
Comparison	Implication	A	B	C	E	
IH5D vs. Ctrl	IH5D	↓ (6/11)	↑ (5/5)	↑ (18/20)	↑ (11/11)	62385
IH28D vs. Ctrl28	IH28D	↓ (6/11)	↑ (5/5)	↑ (10/20)	↑ (11/11)	
12 m vs. Ctrl	Aging	↓ (11/11)	↑ (5/5)	↑ (18/20)	↑ (11/11)	
18 m vs. 3.5 m	Aging	− (0/11)	− (1/5)	→ (8/20)	− (0/11)	29075
KA vs. Saline	KA treatment	− (4/11)	↑ (5/5)	↑ (20/20)	↑ (11/11)	60772
Dicer KO vs. Ctrl	Dicer ablation	↓ (6/11)	− (0/5)	→ (8/20)	− (0/11)	61937
Dicer KO vs. Ctrl	Dicer ablation	− (1/11)	− (0/5)	→ (8/20)	↑ (8/11)	23847
Glud1 Tg vs. Ctrl	Moderate glutamate excess	− (0/11)	↑ (5/5)	↑ (16/20)	− (1/11)	11419

Shown are alteration patterns of GO term clusters A, B, C and E in each experimental model. An up arrow (a down arrow) was indicated if 50% or more of the GOs belonging to the cluster have a p value of 0.01 or less in the direction of increasing (decreasing) gene expression, respectively. Each arrow was indicated with a description such as “16/20”, which was stated that 16 of the 20 total numbers made significant fluctuations. In addition, we used a right-pointing arrow to represent “8/20”. All the rest parts of the table were indicated using hyphens. Comparisons and their biological implications are shown in the two rows on the left. A corresponding GSE # of each data is shown at the rightmost row. Our experimental data have been deposited as GSE62385. Ctrl, control; IH5D, exposed to IHT for 5 days; IH28D, exposed to IHT for 28 days; m, months; KA, kainic acid; KO, knock out, Glud1, glutamate dehydrogenase 1; Tg, transgenic

The dendrogram (Fig. 2c, bottom; Additional file 1: Figure S1, bottom) also revealed that previous several experimental data had similar comprehensive gene expression to GSE62385 (IHT or aging) as follows: aging (18 months old vs. 3.5 months old) (GSE29075) [32], kainic acid (KA) treatment (KA-treated mice vs. Saline-treated mice) (GSE60772) [33], Dicer ablation (Dicer knock out (KO) mice vs. Ctrl) (GSE23847, GSE61937) [34, 35], and moderate glutamate excess (glutamate dehydrogenase 1 (Glud1) transgenic (Tg) mice vs. Ctrl) (GSE11419) [36, 37]. In particular, the alteration patterns of clusters A, B, C and E in these experimental models are summarized in Table 1. The left side of Fig. 2c displayed examples of data (GSE57583, GSE21137) [38, 39], in which the alteration patterns of GO terms differed from our experimental data (GSE62385).

### IHT increased tau phosphorylation

In order to provide more functional information, we mapped and visualized genes that were altered by IHT or aging on the KEGG PATHWAY database. Previous studies have reported that the balances of kinases and/or phosphatases activities are altered during aging [40, 41]. Therefore, to determine whether imbalances of kinases and/or phosphatases occur with IHT or aging, we searched “MAPK signaling pathway” (map04010) (Additional file 1: Figure S2) and “PI3K-Akt signaling pathway” (map04151) (Additional file 1: Figure S3), since both pathways involve various kinases and phosphatases. The extent of alterations was amplified in the order corresponding to IH5D, and IH28D or aging, suggesting that IHT produces imbalances in kinases and/or phosphatases. In particular, we predicted the imbalance might direct towards an increase in kinase activities since the mapping on KEGG PATHWAY database mainly showed alterations in some kind of kinases.

In order to evaluate the imbalances of kinases and/or phosphatases, we focused on the microtubule-associated protein, tau. Tau has more than 40 potential phosphorylation sites, which are targets of various kinases and phosphatases [42, 43]. Altered levels of P-tau are therefore expected to reflect alterations in the balance of kinases and/or phosphatases. In addition, we found that IHT or aging, as well as Dicer KO models, demonstrate similar gene expression (Fig. 2c, the bottom dendrogram), particularly for alterations in several GO terms in cluster C (Fig. 2c; Table 1; Additional file 1: Figure S1). Furthermore, Dicer KO mice have exhibited increased P-tau [35, 44]. Therefore, we investigated whether IHT resulted in altered level of P-tau.

**Fig. 3:**
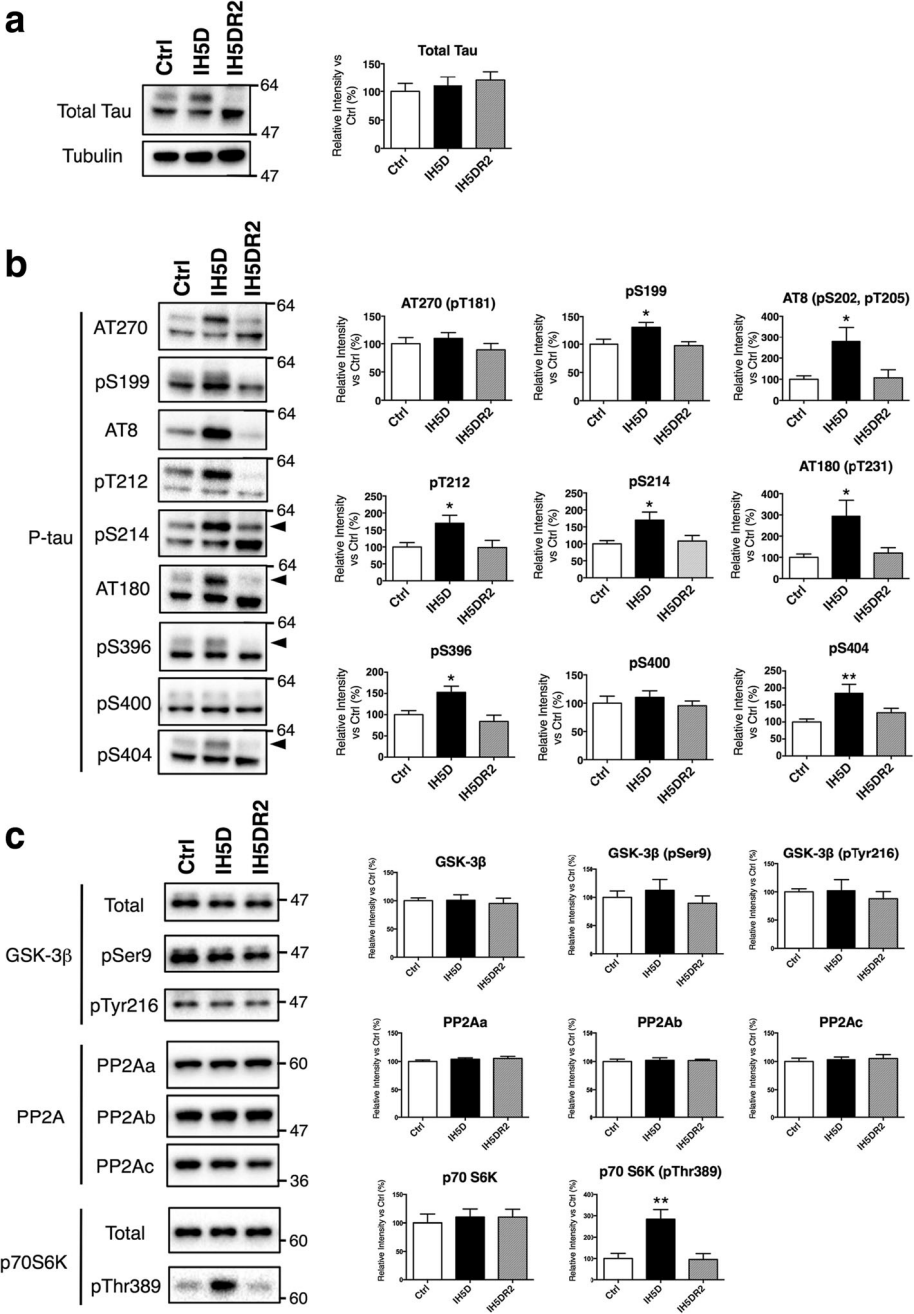
Effects of IH5D and IH5DR2 on tau phosphorylation and its related enzymes. **a** Total tau was not altered between the Ctrl, IH5D, and IH5DR2 groups. Representative blot for total tau or α-tubulin are shown (left panels), as well as quantification (right panels). **b** Intermittent hypoxia treatment (IHT) produced an increase in tau phosphorylation. Representative blots are shown for each epitope (left panels) and quantification data (right panels). All blots indicated phosphorylated tau. Arrowheads indicated quantified bands. Quantification data include the mean + SEM (*n* = 9). * *p* <0.05, ** *p* <0.01 vs. Ctrl. **c** Analyses were performed for enzymes (GSK-3β, PP2A, and p70S6K) related to tau phosphorylation. Representative blots (left panels) and quantification data (right panels) are shown. Quantification data include the mean + SEM (*n* = 5, 6). ** *p* <0.01 vs. Ctrl

We investigated level of P-tau in Ctrl, IH5D, and IH5DR2 groups by using TBS-soluble hippocampal fractions. Total tau levels were similar between Ctrl, IH5D, and IH5DR2 groups (Fig. 3a). Samples from IH5D mice showed increased phosphorylation at Ser199, Ser202, Thr205, Thr212, Ser214, Thr231, Ser396, and Ser404 epitopes (Fig. 3b, Ctrl vs. IH5D). Normoxia treatment for 2 days reduced P-tau to Ctrl levels (Fig. 3b, Ctrl vs. IH5DR2).

Next, we aimed to understand the mechanisms of altered P-tau. In particular, involvement of mammalian target of rapamycin (mTOR) was suggested using the map “PI3K-Akt signaling pathway” (map04151) in the KEGG PATHWAY database (Additional file 1: Figure S3). Previous reports have suggested that the mTOR signaling pathway may affect P-tau [45, 46]. In order to investigate the role of mTOR signaling in IHT-induced P-tau increases, we focused on p70 S6 kinase (p70S6K), glycogen synthase kinase-3β (GSK-3β), and protein phosphatase 2A (PP2A), which are direct or indirect P-tau regulators downstream of mTOR (Additional file 1: Figure S4) [46, 47]. Total GSK-3β and its phosphorylation at Ser9 and Tyr216 were comparable between the Ctrl, IH5D, and IH5DR2 groups (Fig. 3c, the top panels). PP2A is composed of subunits (i.e., PP2Aa, PP2Ab, and PP2Ac), and amounts of these PP2A subunits were comparable between the Ctrl, IH5D, and IH5DR2 groups (Fig. 3c, the middle panels). While total pS70S6K was comparable between the Ctrl, IH5D, and IH5DR2 groups, phosphorylated p70S6K increased significantly in the IH5D group (Fig. 3c, the bottom panels, Ctrl vs. IH5D). Normoxia treatment for 2 days reduced phosphorylated p70S6K to Ctrl levels (Fig. 3c, the bottom panels, Ctrl vs. IH5DR2). Increased phosphorylation of p70S6K indicates mTOR signaling pathway activation [45]. Therefore, the results suggest that IHT increased P-tau via at least activation of mTOR signaling pathway. In contrast, the effects of IH5D were reversed by normoxia treatment.

**Fig. 4:**
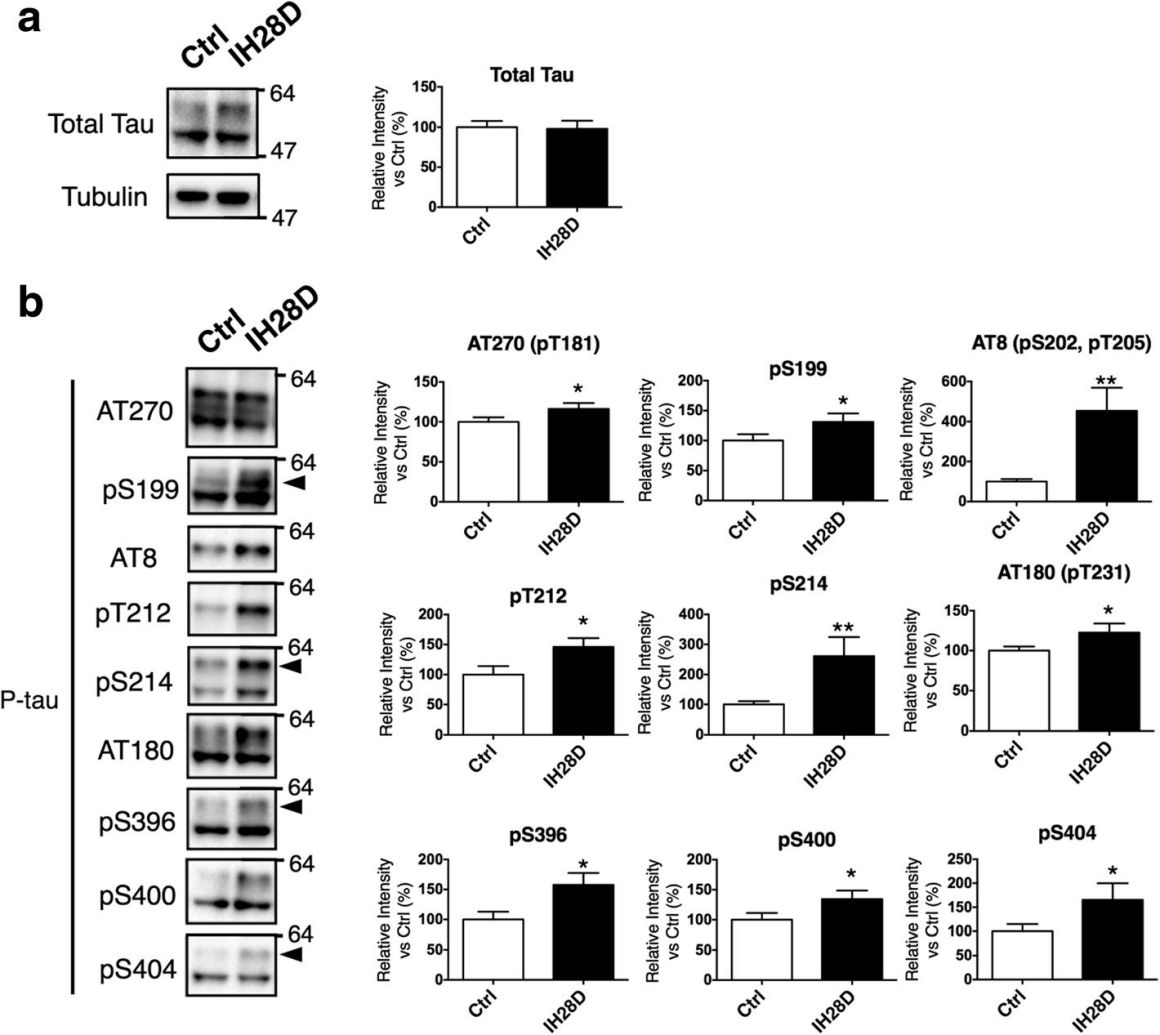
IH28D increases tau phosphorylation. **a** Total tau did not differ between the Ctrl and IH28D groups. Representative blots for total tau or α-tubulin are shown (left panels), as well as quantification data (right panels). **b** Representative blots for each epitope. All blots indicated phosphorylated tau. Arrowheads indicate quantified bands. Quantification data include the mean + SEM (*n* = 12). * *p* <0.05, ** *p* <0.01 vs. Ctrl. Abbreviations: Ctrl = reared at our institution for 28 days, IH28D = exposed to IHT for 28 days

Finally, we investigated whether elongation of the IHT periods to 28 days affected P-tau. Total tau level was comparable between the Ctrl and IH28D groups (Fig. 4a). The IH28D group had increased P-tau at Thr181, Ser199, Ser202, Thr205, Thr212, Ser214, Thr231, Ser396, Ser400, and Ser404 epitopes (Fig. 4b). Compared to the result of the IH5D group, we detected additional phosphorylated tau sites at Thr181 and Ser400 in the IH28D group. Elongation of the IHT period therefore promoted tau phosphorylation.

### Effect of IHT on behavior in Y-maze tests

**Fig. 5:**
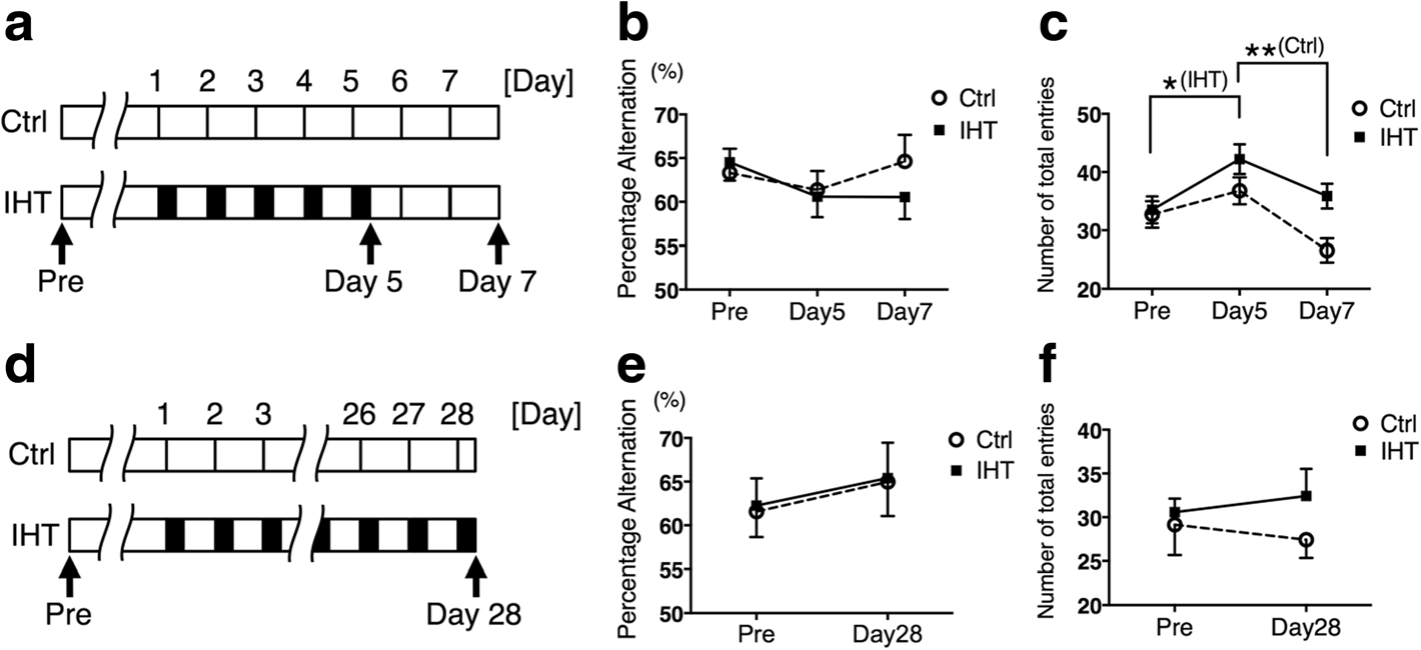
Effects of intermittent hypoxia treatment (IHT) on behavior on Y-maze tests. **a-c** Effect of IH5D on the Y-maze test. **a** Schedule of Y-maze tests. Black boxes indicate IHT, and white boxes indicate normoxia treatment. **b** Percentage alternation is shown by the broken line with open circles (Ctrl) and by the solid line with closed squares (IHT). Data include the mean + SEM (Ctrl, *n* = 14) and mean - SEM (IHT, *n* = 14). **c** Number of total entries is shown by the broken line with open circles (Ctrl) and by the solid line with closed squares (IHT). Data include the mean ± SEM (Ctrl, *n* = 14; IHT, *n* = 14). * *p* <0.05, ** *p* <0.01. Abbreviations: Ctrl = control group, IH5D = exposed to IHT for 5 days, IH5DR2 = exposed to IHT for 5 days, followed by normoxia treatment for 2 days. **d-f** Effect of IH28D on the Y-maze test. **d** Schedule of Y-maze tests. Black boxes indicate IHT, and white boxes indicate normoxia treatment. **e** Percentage alternation is shown by the broken line with open circles (Ctrl) and by the solid line with closed squares (IHT). Data include the mean - SEM (Ctrl, *n* = 7) and mean + SEM (IHT, *n* = 7). **f** Number of total entries is shown by the broken line with open circles (Ctrl) and by the solid line with closed squares (IHT). Data include the mean - SEM (Ctrl, *n* = 7) and mean + SEM (IHT, *n* = 7). Abbreviations: Ctrl = reared at our institution for 28 days, IH28D = exposed to IHT for 28 days

Accumulating evidence suggest that the level of P-tau correlates with aging or AD-associated cognitive impairments [48–53]. In order to investigate whether the IHT, including an increase in P-tau, affected behavior, we performed Y-maze tests. Mice were subjected to the IH5DR2 protocols, and compared with Ctrl. The tests were performed at day 0 (before any exposure; Pre), day 5, and day 7 (Fig. 5a). Percentage alternation results indicated no impairments in working memory (Fig. 5b). The number of total entries increased significantly at day 5 compared to Pre in the IHT group, indicating hyperactivity (Fig. 5c). We also detected a decrease in the number of total entries at day 7 compared to day 5 in the Ctrl group (Fig. 5c), suggesting habituation to repeated Y-maze tests in the Ctrl group, but not in the IHT group. The increase in P-tau may be related to the behavioral effects.

Next, we performed Y-maze tests using mice exposed to the IH28D protocols. The tests were performed at day 0 (before any exposure; Pre), and day 28 (Fig. 5d). Percentage alternation results indicated that the IH28D group did not show significant impairments in working memory (Fig. 5e). The number of total entries still showed a tendency toward hyperactivity in the IH28D group, but the difference was not significant (Fig. 5f).

### IH28D, but not IH5D, results in altered postsynaptic proteins

**Fig. 6:**
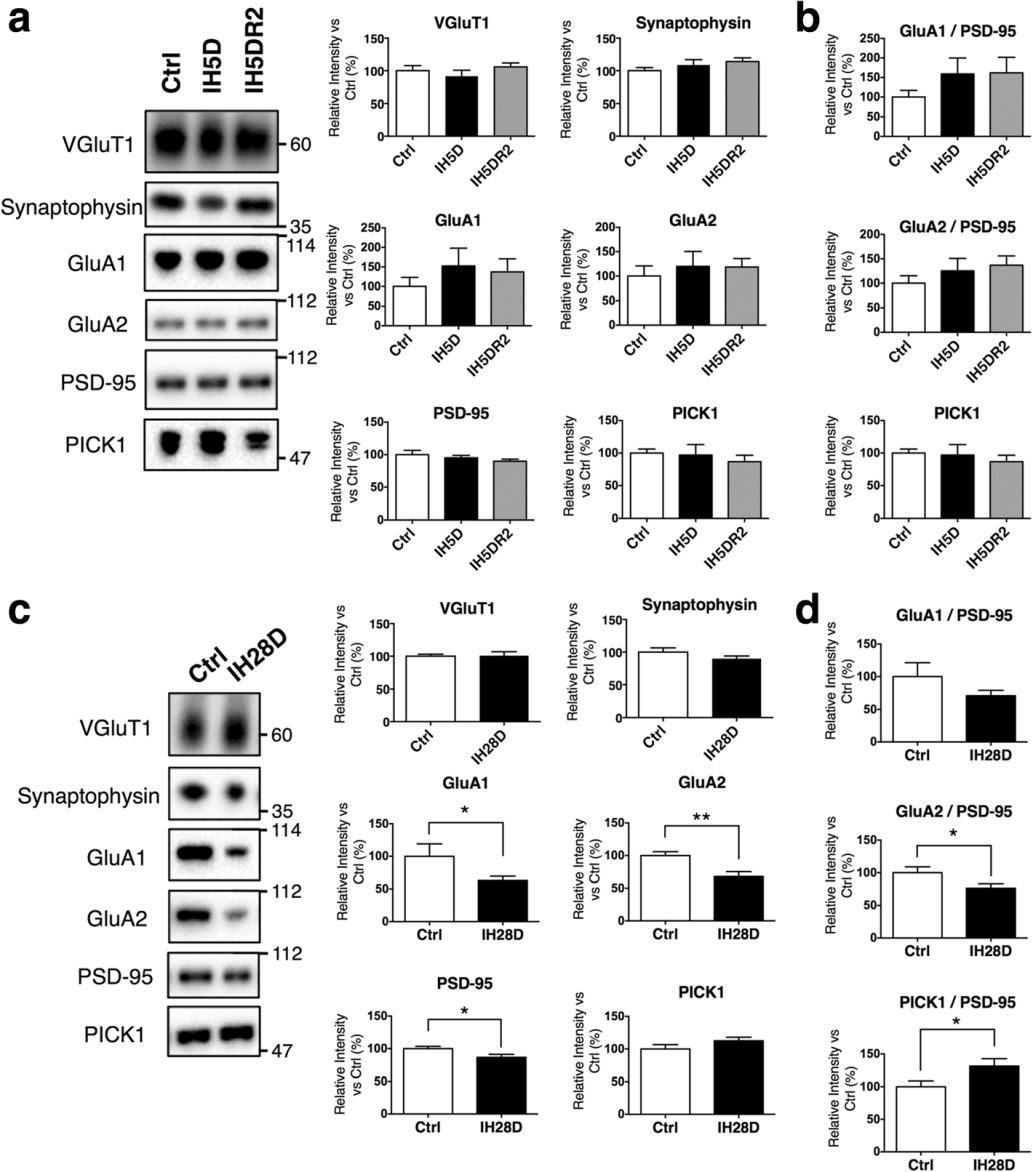
Effects of intermittent hypoxia treatment (IHT) on pre- and post-synaptic proteins. **a** Effect of IH5D and IH5DR2 on levels of VGluT1, synaptophysin, GluA1, GluA2, PSD-95, and PICK1. Representative blots are shown for hippocampal TBS-insoluble fraction (left panels). Quantification data include the mean + SEM (*n* = 6) (right panels). **b** Quantification data for each postsynaptic protein in relation to PSD-95 in (**a**). Abbreviations: Ctrl = control group, IH5D = exposed to IHT for 5 days, IH5DR2 = exposed to IHT for 5 days, followed by normoxia treatment for 2 days. **c** Effect of IH28D on levels of VGluT1, synaptophysin, GluA1, GluA2, PSD-95, and PICK1. Representative blots are shown for hippocampal TBS-insoluble fractions (left panels). Quantification data include the mean + SEM (*n* = 7) (right panels). **d** Quantification data for each postsynaptic protein in relation to PSD-95 in (**c**). * *p* <0.05, ** *p* <0.01 vs. Ctrl. Abbreviations: Ctrl = reared at our institution for 28 days, IH28D = exposed to IHT for 28 days

The GO-based microarray analysis also revealed similarities between IHT (GSE62385), aging (GSE62385, GSE29075), KA treatment (GSE60772), and moderate glutamate excess (GSE11419). KA stimulates glutamate-relevant pathways, thereby producing excitatory cell death [54]. We investigated the possibility that IHT and/or aging also alter glutamate pathways, using a map “glutamatergic synapse” in the KEGG PATHWAY database (map04724) (Additional file 1: Figure S5). The extent of alteration was progressively amplified in the order corresponding to IH5D, IH28D and aging, suggesting that IHT alters synaptic proteins. Therefore, the TBS-insoluble fraction of our hippocampal samples was used for biochemical analyses of presynaptic (VGluT1, synaptophysin) and postsynaptic (GluA1, GluA2, PSD-95, and PICK1) proteins. In particular, VGluT1, GluA1, GluA2, and PICK1 were closely related to glutamatergic synapse. The levels of pre- and postsynaptic proteins were not altered between the Ctrl, IH5D, and IH5DR2 groups (Fig. 6a). Next, we evaluated the ratio of between each postsynaptic protein (GluA1, GluA2, PICK1) to PSD-95. No alterations were observed between the Ctrl, IH5D, and IH5DR2 groups (Fig. 6b). Presynaptic proteins (VGluT1, synaptophysin) were not altered in the IH28D group (Fig. 6c). In contrast, IH28D reduced postsynaptic proteins (GluA1, GluA2, and PSD-95), with the exception of PICK1. The ratios of each postsynaptic protein (GluA1, GluA2, PICK1) to PSD-95 were as follows: GluA1 decreased but the difference was not significant, GluA2 significantly decreased, and PICK1 significantly increased (Fig. 6d). Elongation of IHT produced a decrease in the levels of postsynaptic proteins, and altered the ratio of postsynaptic proteins to PSD-95.

In summary, our in vivo observations (Figs. 3, 4, 5 and 6) are consistent with shared biological processes between IHT and aging (Fig. 2), thereby elucidating potential mechanisms of IHT-induced cognitive impairments that have been previously reported.

## Discussion

### Common biological processes between IHT and aging may be related to AD

We firstly performed PCA and GO-based microarray analyses in order to elucidate the effects of IHT on hippocampal functioning. Although various studies have used microarray analyses so far, it is not easy to extract biological meanings from the analyses. In this study, we applied the previously-established GO-based microarray analysis [25] to the present study, and successfully found that IHT and aging shared alterations in some common GO, which were also observed with kainic acid treatment, Dicer ablation, or moderate glutamate excess. By performing in vivo analyses, we found that IHT increased P-tau via at least activation of mTOR signaling pathway, and decreased levels of postsynaptic proteins. These results confirmed the validity of our in silico analyses. The present study also support a previous suggestion that intermittent hypoxia is a cause of aging [31].

Aging is a major risk factor for AD [55]. With aging, the brain may become susceptible to AD via amplification of molecular alterations that are qualitatively similar to normal aging [56, 57]. Given that IHT shared common biological processes to aging, it is possible that IHT also shared common biological processes to those involved in AD onset. During normal aging, and to a greater extent in tauopathies including AD, tau is phosphorylated to form neurofibrillary tangles (NFTs) in the brain [43, 57–62]. Our GO-based microarray analysis revealed that Dicer ablation, KA treatment, and a moderate glutamate excess produce similar gene expression patterns to IHT or aging (Fig. 2; Additional file 1: Figure S1). An increase in P-tau was observed in our IHT model (Figs. 3 and 4), and was also reported previously in Dicer KO mice [35] and KA-treated mice [63]. Dicer ablation could may alter GO terms related to RNA metabolism, since Dicer is a type III RNase enzyme responsible for processing microRNA, which degrades or represses mRNA translation [64]. Indeed, Dicer ablation alters several GO terms in cluster C that were related to RNA metabolism (Table 1; Fig. 2c; Additional file 1: Figure S1). Recent findings also indicate that alteration in microRNA metabolism may increase P-tau [65, 66], suggesting involvement of alterations in the balance of kinases and/or phosphatases. Alterations to clusters B and C also occur with KA treatment, and a moderate glutamate excess (Table 1). KA produces excitatory cell death [54], and Glud1 Tg mice exhibit neuronal loss in select brain regions, concurrent with indications of accelerated aging [36, 37]. In other words, both KA-treated mice and Glud1 Tg mice exhibit excessive neuronal excitation, which has been indicated as a common mechanism underlying aging or neurodegenerative diseases such as AD [67].

Accumulation of NFTs is correlated with neuronal loss and cognitive impairments in AD brains [48, 49]. These accumulations may also be responsible for memory loss associated with aging and memory deficits in some cases of mild cognitive impairment, which is an intermediate stage between aging and AD [50, 51]. Several mouse models expressing wild-type human tau have exhibited age-dependent accumulation of P-tau [68], which is associated with cognitive impairments [52, 53]. Some AD mouse models also show hyperactivity in Y-maze tests, which is ameliorated by reducing endogenous tau [69, 70]. These findings suggest that increased P-tau is closely relevant to cognitive impairments, including hyperactivity. Indeed, in the present study, hyperactivity in Y-maze tests was observed in the IH5D group (Fig. 5c). But, since the IH28D group did not exhibit a significant hyperactivity (Fig. 5f), the phenotype could be observed in acute phase of IHT rather than chronic phase. These insights suggested that biological processes associated with increment of P-tau are shared by IHT, aging, Dicer ablation, and KA treatment, and are also related to the onset of AD. Therefore, these may explain the mechanisms of the IHT-induced cognitive impairments that have been reported previously [16, 21–23].

We also found that IHT increased phosphorylated p70S6K, which is downstream of mTOR, and may regulate level of P-tau either directly or indirectly, such as through regulating activities of various kinases and/or phosphatases including GSK-3β and PP2A [45, 46] (Additional file 1: Figure S4). Thus, IHT-induced increases in P-tau may be partially explained by mTOR signaling pathway activation. Inhibition of mTOR by rapamycin and a genetically ablation of S6K increased lifespan [71, 72]. Activation of the mTOR signaling pathway has been suggested in relation to AD [24, 73]. Moreover, reducing S6K improved spatial memory and synaptic plasticity in an AD model [74]. Therefore, IHT, aging, and AD may share common biological processes involving the mTOR signaling pathway.

We found that IH28D mice reduced postsynaptic proteins (GluA1, GluA2, PSD-95), but there were no changes in presynaptic proteins (VGluT1, synaptophysin). Our data are consistent with a previous study reporting that mice expressing human wild-type tau had reduced PSD-95, but that synaptophysin was not altered [52]. Recently, it has been suggested that removal of GluA2 from the synapse is involved in the mechanisms of AD. Tau is localized in postsynapse, and is phosphorylated during the removal of GluA2 from synapse [75]. This removal of GluA2 requires interaction between GluA2 and PICK1 [76], which is enhanced by the presence of tau [77]. In the present study, IH28D mice exhibited a decrease in GluA2 and an increase in PICK1 to PSD-95 (Fig. 6), suggesting promotion of GluA2 removal.

These considerations confirmed that IHT and aging share common biological processes, which are also related to AD. However, the precise mechanism linking AD and aging is unknown, perhaps because there have been few models that reflect human-like pathology reflecting the effect of aging. IHT may mimic an environmental stimulus that contributes to AD progression, and may therefore be useful in producing feature that more closely models AD. Applying IHT to the existing AD models, such as an *APP* knock-in model [78], will therefore likely contribute to developing improved AD models that are desired for the future research [79].

### IHT is a novel model to evaluate the physiological roles of P-tau

It still remains an enigma that the precise processes leading to increase in P-tau, as well as the effects of increased P-tau on neurons, the brain, and behavior. Further understanding will be enabled by generating animal models with increased *endogenous* P-tau, without using gene manipulation techniques. Previous studies have reported that non-transgenic mice exposed to starvation [80] or anesthesia [81] have increased endogenous P-tau. However, these previous models are not suitable for long-term observation or behavioral analyses. In contrast, IHT increased *endogenous* P-tau (Figs. 3 and 4), and it is possible to perform behavioral analyses on IHT model mice (Fig. 5). To the best of our knowledge, the IHT model is the first non-transgenic model that enables behavioral analyses under conditions of increased *endogenous* P-tau.

Using the IHT model, we found evidence for activation of the mTOR signaling pathway as a candidate mechanism of increased P-tau in vivo. In addition, our findings support that the increase in P-tau may contribute to hyperactivity in Y-maze tests, which is an AD model-related behavior. Therefore, the IHT model is suitable model for use in studying about the physiological roles of P-tau.

## Additional file

Additional file 1:
**Figure S1.** Complete heatmap generated from the gene ontology (GO)-based microarray analysis. GO heatmap with a gene clustering dendrogram on the left, and a sample correlation dendrogram on the bottom of the heatmap. The heatmap was generated across all samples, including data from our experiment and GEO; *p* values were calculated by the hypergeometric test and denoted by -log_10_ *P*; overrepresented GO terms are shown on the right and left in one pairwise comparison group, and are also shown in red and blue (increased (+) and decreased (−), respectively). The red-colored dendrogram indicates our data (GSE62385) and experimental data located near our data. The green rectangles indicate the parts that are displayed as Fig. 2. **Figures S2, S3, and S5.** Mapping results of GO-based microarray analysis using the Kyoto Encyclopedia of Genes and Genomes (KEGG) PATHWAY database. Altered genes in IH5D, IH28D,and 12 m compared to their control were mapped and visualized on the map04010 (MAPK signaling pathway) (Fig. S2), map04151 (PI3K-Akt signaling pathway) (Fig. S3), and the map04724 (glutamatergic synapse) (Fig. S5). Differentiation ratios (*r*) are visualized by red (2 < *r* < infinity) or blue (0 < *r* < 0.5). Abbreviations: IH5D = exposed to IHT for 5 days, IH28D = exposed to IHT for 28 days, 12 m = reared at our institution for 12 months. **Figure S4.** Schematic of mTOR-related enzymes that regulate tau phosphorylation. mTOR activates p70S6K, which activates GSK-3β via several factors such as PI3K or Akt. Conversely, mTOR inhibits PP2A. Tau can be phosphorylated by p70S6K or GSK-3β, and dephosphorylated by PP2A. PP2A dephosphorylates Ser9 of GSK-3β, resulting in GSK-3β activation. (8.79 mb)

## Abbreviations

AD: Alzheimer disease
Ctrl: Control
FIO_2_: Fraction of inspired O_2_
GEO: Gene Expression Omnibus
Glud1: Glutamate dehydrogenase 1
GO: Gene ontology
GSK-3β: Glycogen synthase kinase-3β
IHT: Intermittent hypoxia treatment
KA: Kainic acid
KEGG: Kyoto Encyclopedia of Genes and Genomes
KO: Knock out
M: Months
mTOR: Mammalian target of rapamycin
NFT: Neurofibrillary tangles
p70S6K: p70 S6 kinase
PCA: Principal component analysis
PP2A: Protein phosphatase 2A
P-tau: Phosphorylated tau
SDB: Sleep-disordered breathing
TBS: Tris-buffered saline
Tg: Transgenic
