# Supplementary material for: Treatment of intermittent hypoxia increases phosphorylated tau in the hippocampus via biological processes common to aging
Source: Mol Brain. 2017 Jan 5;10:2. doi: 10.1186/s13041-016-0282-7 (PMC5217192; doi:10.1186/s13041-016-0282-7)
Supplement: Additional file 1: — Figure S1. Complete heatmap generated from the gene ontology (GO)-based microarray analysis. GO heatmap with a gene clustering dendrogram on the left, and a sample correlation dendrogram on the bottom of the heatmap. The heatmap was generated across all samples, including data from our experiment and GEO; p values were calculated by the hypergeometric test and denoted by -log10 P; overrepresented GO terms are shown on the right and left in one pairwise comparison group, and are also shown in red and blue (increased (+) and decreased (−), respectively). The red-colored dendrogram indicates our data (GSE62385) and experimental data located near our data. The green rectangles indicate the parts that are displayed as Fig. 2. Figures S2, S3, and S5. Mapping results of GO-based microarray analysis using the Kyoto Encyclopedia of Genes and Genomes (KEGG) PATHWAY database. Altered genes in IH5D, IH28D,and 12 m compared to their control were mapped and visualized on the map04010 (MAPK signaling pathway) (Fig. S2), map04151 (PI3K-Akt signaling pathway) (Fig. S3), and the map04724 (glutamatergic synapse) (Fig. S5). Differentiation ratios (r) are visualized by red (2 < r < infinity) or blue (0 < r < 0.5). Abbreviations: IH5D = exposed to IHT for 5 days, IH28D = exposed to IHT for 28 days, 12 m = reared at our institution for 12 months. Figure S4. Schematic of mTOR-related enzymes that regulate tau phosphorylation. mTOR activates p70S6K, which activates GSK-3β via several factors such as PI3K or Akt. Conversely, mTOR inhibits PP2A. Tau can be phosphorylated by p70S6K or GSK-3β, and dephosphorylated by PP2A. PP2A dephosphorylates Ser9 of GSK-3β, resulting in GSK-3β activation. (8.79 mb) [file 13041_2016_282_MOESM1_ESM.pdf]

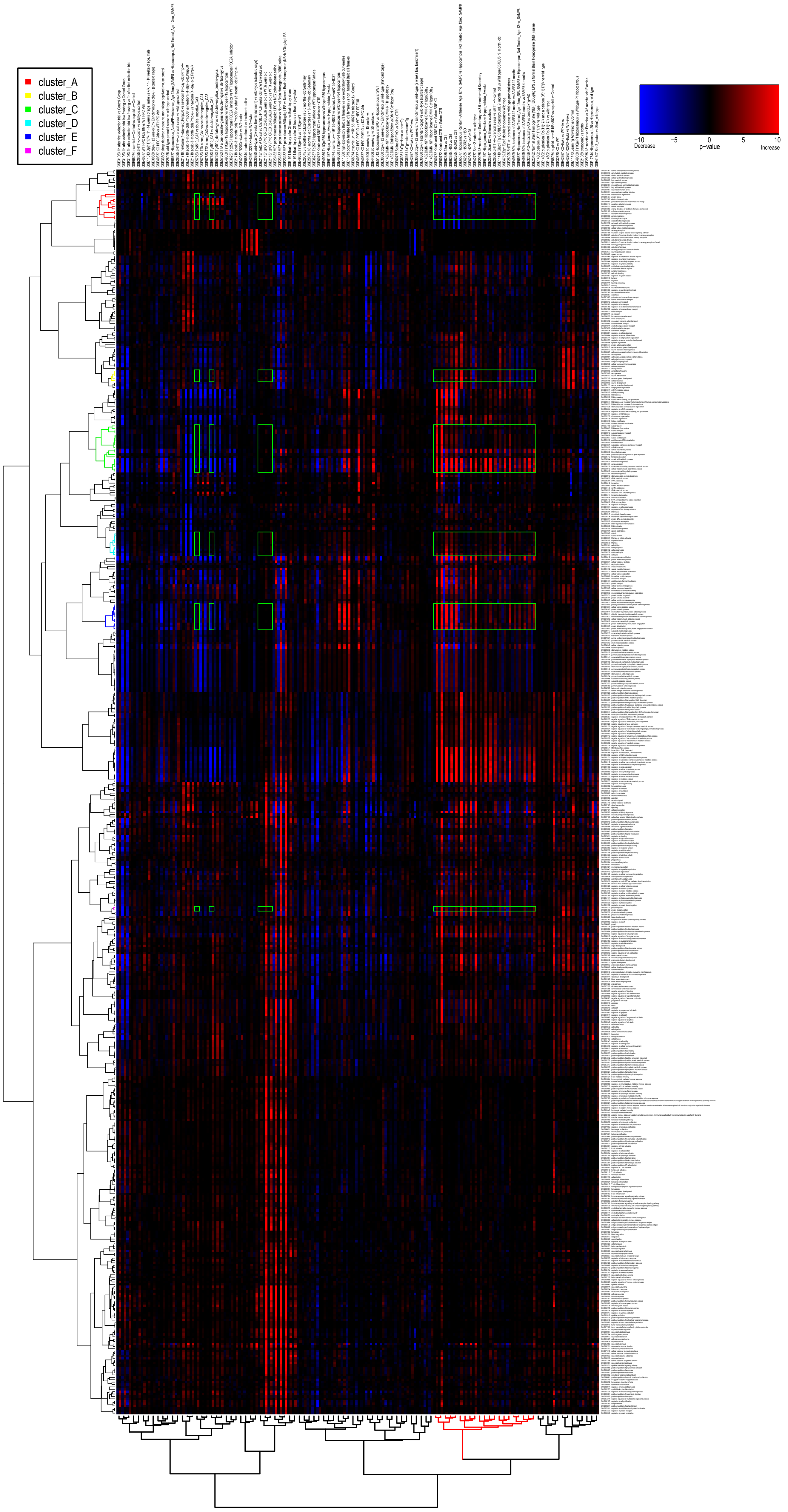
cluster_A
cluster_B
cluster_C
cluster_D
cluster_E
cluster_F
−10
−5
0
5
10
Decrease
p−value
Increase

Figure S1

MAPK signaling pathway (map04010)

IH5D

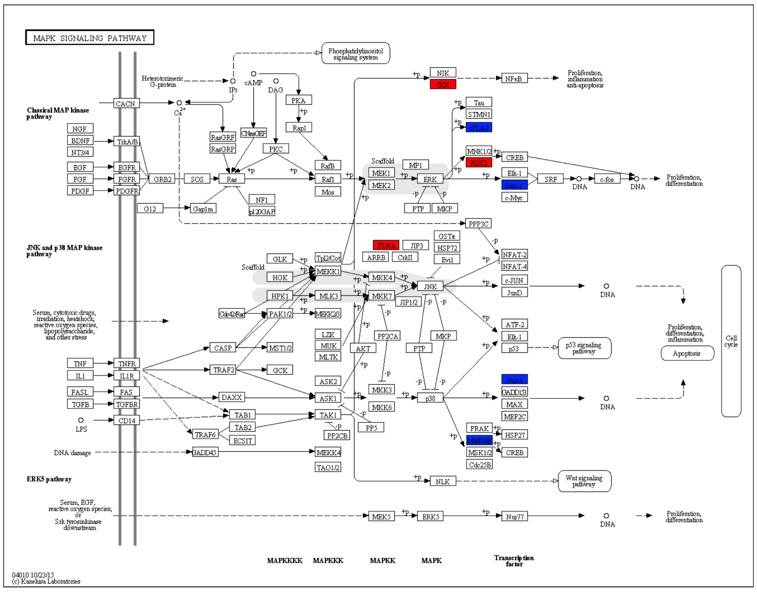

IH28D

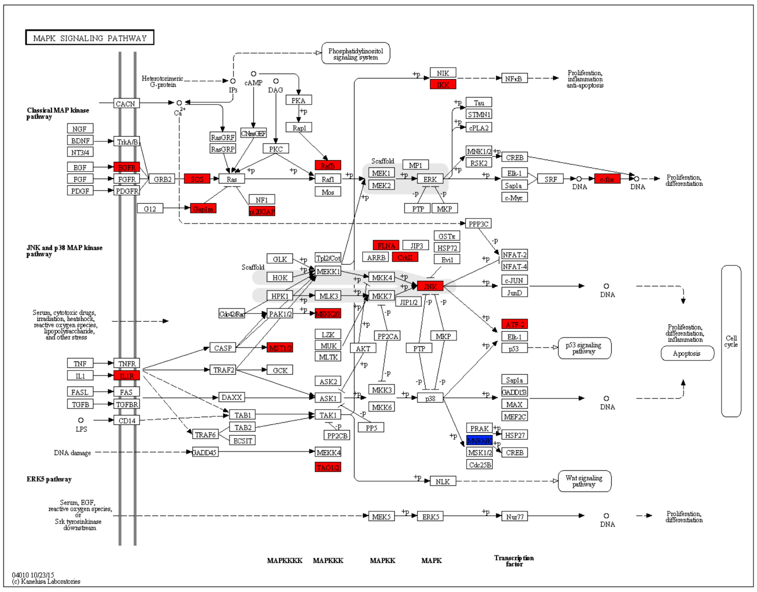

12m

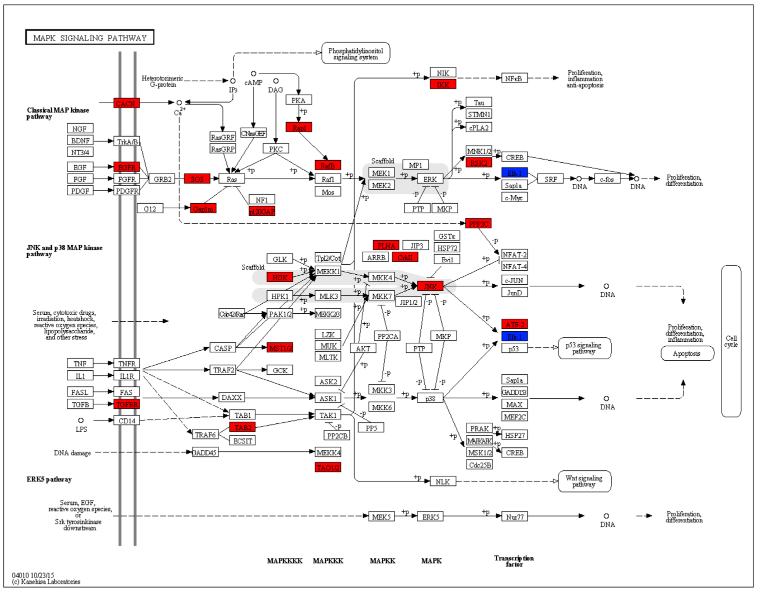

Figure S2

PI3K-Akt signaling pathway (map04151)

IH5D

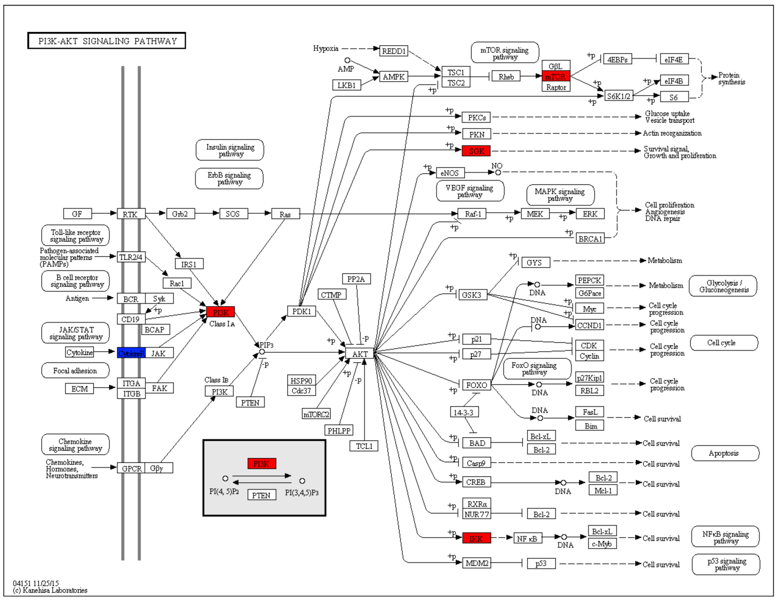

IH28D

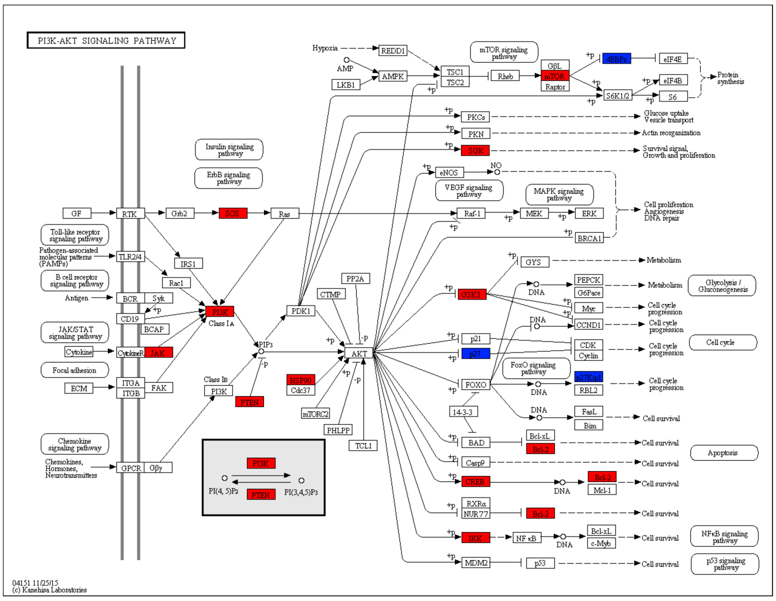

12m

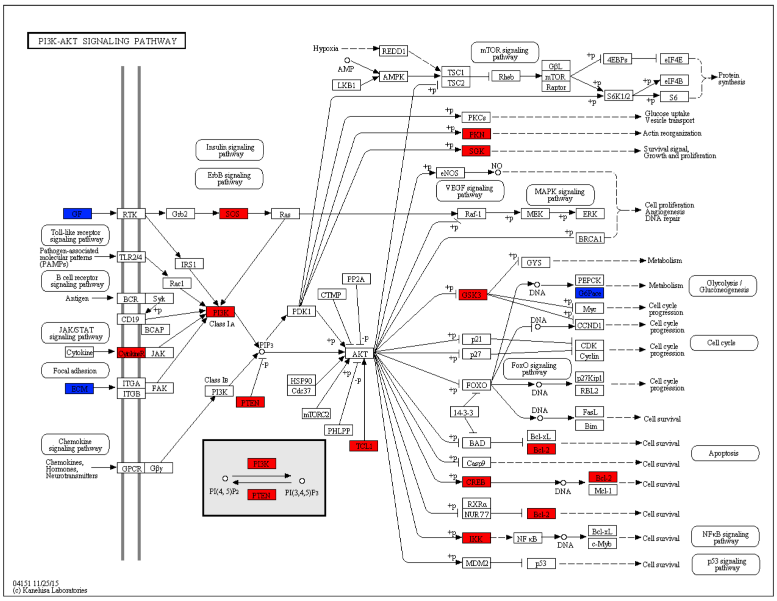

Figure S3

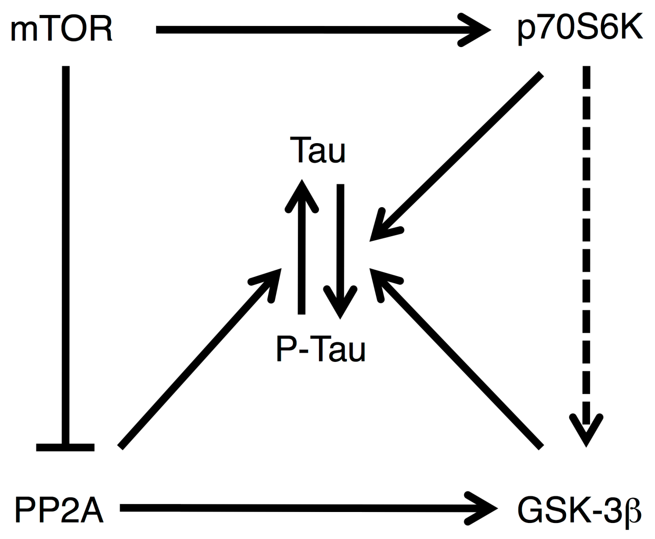

Figure S4

Glutamatergic Synapse (map04724)

IH5D

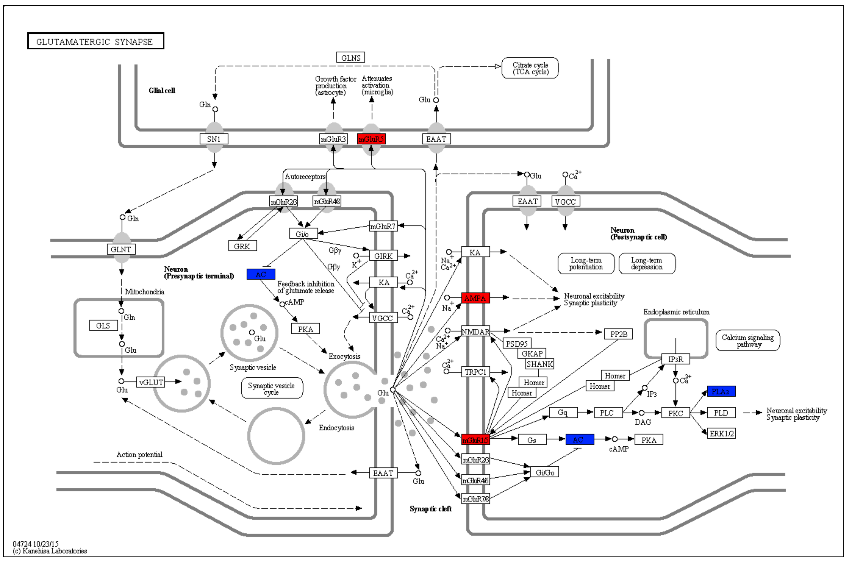

IH28D

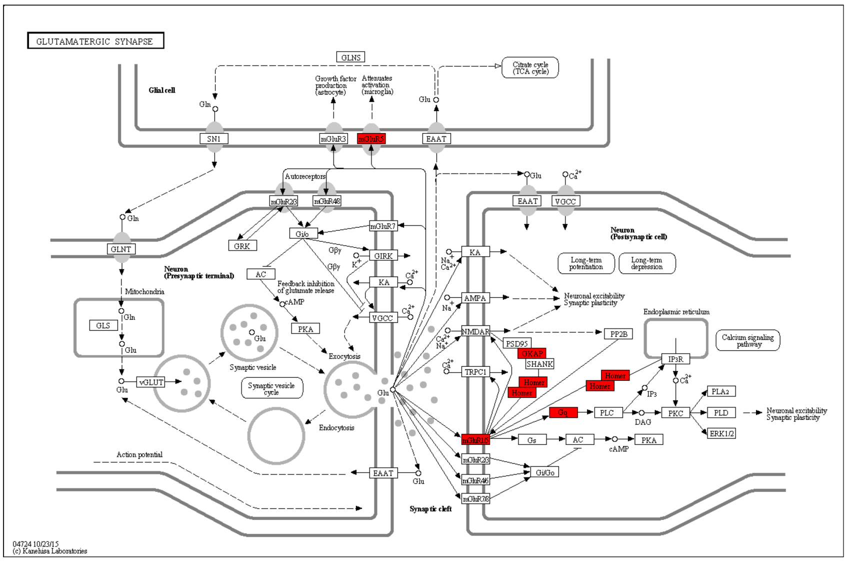

12m

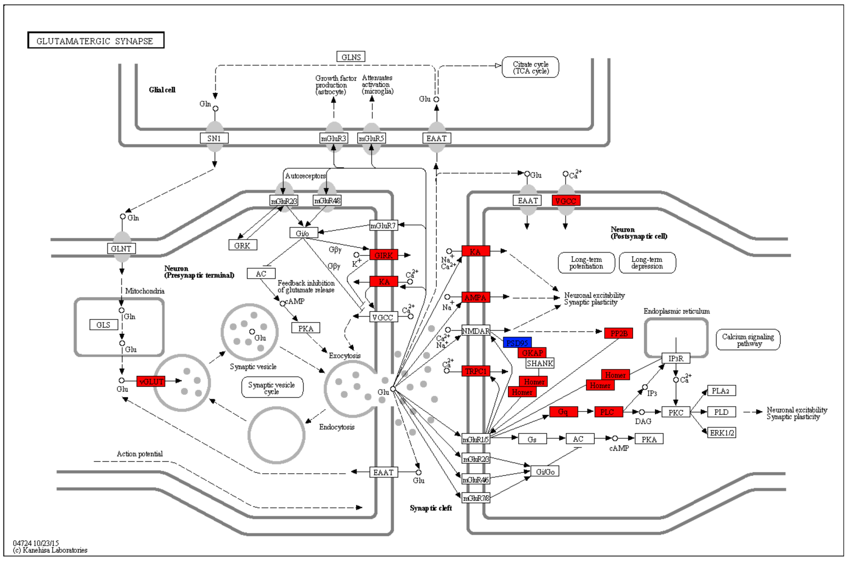

Figure S5
